# Bedside bronchoscopy in intubated patients with acute exacerbation of COPD requiring invasive mechanical ventilation: clinical applications, procedural timing, and standardized management

**DOI:** 10.3389/fmed.2026.1948282

**Published:** 2026-09-01

**Authors:** Juan He, Yu Yao, Xing Chen, Yanyan Yang, Wen Tao

**Affiliations:** Department of Respiratory and Critical Care Medicine, Pengshan District People’s Hospital, Meishan, China

**Keywords:** acute exacerbation of chronic obstructive pulmonary disease, airway secretion clearance, bedside bronchoscopy, mechanical ventilation, procedural timing, pulmonary infection control window, standardized management

## Abstract

**Introduction:**

Bedside bronchoscopy is a valuable diagnostic and therapeutic tool for intubated patients with acute exacerbation of chronic obstructive pulmonary disease (AECOPD) receiving invasive mechanical ventilation, yet evidence-based guidance on procedural timing remains limited.

**Methods:**

This narrative review synthesized evidence from guidelines, systematic reviews, randomized controlled trials, and clinical studies identified through PubMed, Cochrane Library, and Chinese databases (CNKI, Wanfang) from inception through June 2026. Selection was iterative and based on relevance to the four clinical scenarios examined.

**Results:**

The available evidence suggests that bedside bronchoscopy may be associated with improved oxygenation, reduced ventilator days, lower VAP rates, and facilitated weaning. We propose a preliminary four-scenario framework for procedural timing: early post-intubation, during mechanical ventilation, pre-extubation (at the proposed pulmonary infection control window), and post-extubation failure. Key aspects of standardized management—including pre-procedural risk assessment, ventilator adjustments, sedation strategies, intraprocedural monitoring, and post-procedural care—are discussed, with special attention to resource constraints in secondary hospital ICUs.

**Discussion:**

The evidence base remains limited and predominantly derived from small, single-center studies, many with potential risk of bias. This framework is hypothesis-generating and requires prospective validation before informing clinical practice recommendations. Future pragmatic multicenter randomized trials are needed.

## 1 Background

In intubated AECOPD patients with unexplained desaturation despite a patent endotracheal tube, the differential diagnosis is broad and includes tube malposition, pneumothorax, mucus plugging, dynamic hyperinflation, ventilator-associated pneumonia, and airway collapse. After tube position and patency have been verified, bedside bronchoscopy can directly identify and manage the airway-related causes—specifically mucus plugging, airway assessment, and endoluminal abnormalities—whereas other causes require additional diagnostic modalities.

Bedside bronchoscopy can provide a direct answer, yet the optimal timing for its performance remains a matter of clinical judgment rather than evidence-based determination.

Bedside bronchoscopy can directly visualize the airway, target segmental mucus plugs, and obtain lower respiratory tract specimens—all without transporting a critically ill patient outside the ICU. Uluc et al. (1) analyzed 332 fiberoptic bronchoscopy (FOB) procedures in a respiratory ICU, confirming BAL specimen collection (43.6%) and airway secretion clearance (30.4%) as the two most common indications. Scala (2) characterized these procedures as valuable adjuncts in the diagnostic and therapeutic pathway of critically ill patients.

The unresolved question—and the one this review addresses—is timing: at what point in the course of mechanical ventilation should bronchoscopy be performed? No guideline currently specifies the optimal timing—early after intubation, during maintenance ventilation, at the transition to extubation, or after extubation failure. This gap is particularly relevant for secondary hospital ICUs, where access to bronchoscopy and staff training differ markedly from tertiary referral centers. This review examines the clinical evidence for each application and proposes a timing framework based on available data.

**Figure 1:**
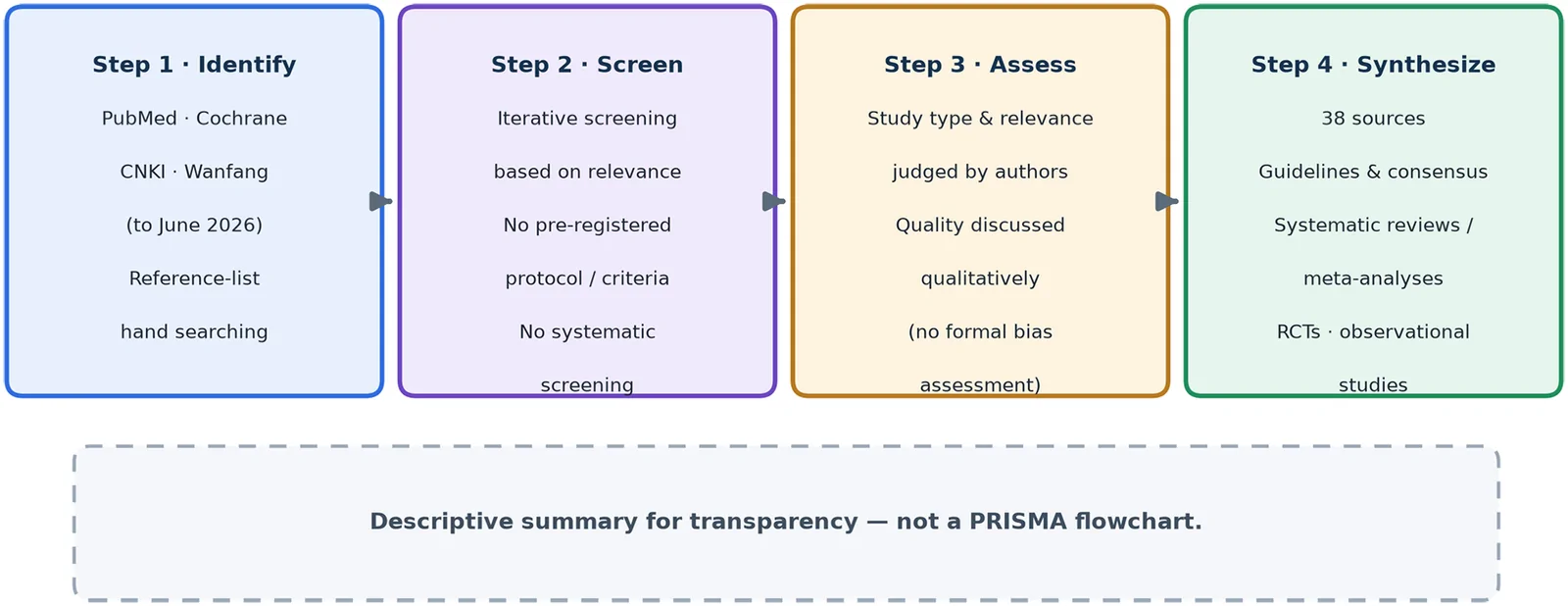
Simplified overview of the literature identification and selection process. This diagram is descriptive and is not a PRISMA flowchart; it reflects the narrative review design (no pre-registered protocol, no predefined inclusion/exclusion criteria, and no systematic screening of retrieved records).

Scope and search method: This is a narrative review. We conducted a targeted literature search of PubMed, Cochrane Library, CNKI, and Wanfang databases from inception through June 2026, supplemented by hand-searching of reference lists of retrieved articles. Given the narrative design of this review, no protocol was pre-registered, no predefined inclusion or exclusion criteria were applied, and records were not systematically screened; selection was iterative and based on relevance to the four clinical scenarios examined. We synthesized evidence from clinical guidelines, systematic reviews and meta-analyses, randomized controlled trials, and clinical studies judged by the authors to be most relevant to each topic area, with methodological quality discussed qualitatively throughout the text. Conference abstracts, case reports, and non-peer-reviewed sources were not prioritized as primary evidence sources, although they were not systematically excluded through a formal screening process. Search terms included combinations of the following: acute exacerbation of COPD, bronchoscopy, bronchoalveolar lavage, mechanical ventilation, weaning, sputum suction, and ventilator-associated pneumonia. For transparency, a simplified, descriptive flow diagram of the literature identification and selection process is provided in Figure 1; this is a descriptive overview and is not a PRISMA flowchart, consistent with the narrative design of this review.

This review aims to: (1) synthesize the available evidence on diagnostic and therapeutic applications of bedside bronchoscopy in mechanically ventilated AECOPD patients; (2) propose a preliminary timing framework based on the proposed PIC window concept; and (3) highlight evidence gaps and resource-specific considerations for secondary hospital ICUs.

## 2 Clinical applications of bedside bronchoscopy

### 2.1 Diagnostic applications

#### 2.1.1 Airway assessment

Bronchoscopy directly shows the tracheobronchial tree—mucosal erythema, edema, ulceration, secretion characteristics, and endoluminal abnormalities such as stenosis, tracheomalacia, or neoplasms that may be missed on conventional imaging (1).

#### 2.1.2 Microbiological sampling

Lower respiratory tract specimens obtained via protected specimen brush (PSB) or BAL yield superior diagnostic accuracy compared with routine sputum cultures in mechanically ventilated patients. Fagon and Chastre (3) established that BAL is essential for diagnosing ventilator-associated pneumonia (VAP). The British Thoracic Society (BTS) guideline (4) and major narrative reviews recommend bronchoscopic sampling when non-invasive methods fail to identify pathogens (5, 6). The diagnostic performance and limitations of various sampling techniques for VAP have been extensively reviewed (7, 8). More recent guidelines, including the IDSA/ATS 2016 clinical practice guideline, recommend non-invasive tracheal aspirate sampling as the preferred initial diagnostic approach for VAP, reserving bronchoscopic BAL for cases where antibiotic de-escalation is planned, suspected multidrug-resistant or unusual pathogens are present, or non-invasive testing remains inconclusive (9).

#### 2.1.3 Differential diagnosis

When mechanically ventilated AECOPD patients develop unexplained oxygenation deterioration or ventilatory failure, bronchoscopy helps differentiate between mucus plugging, airway stenosis, active hemorrhage, aspiration, and other etiologies.

### 2.2 Therapeutic applications

#### 2.2.1 Airway secretion clearance

Airway secretion management follows a stepwise approach. Conventional closed suction catheters, chest physiotherapy, and mucoactive agents (e.g., nebulized N-acetylcysteine or bronchodilators) constitute first-line therapy and are effective in most patients. Bronchoscopy is reserved as a rescue therapy for selected patients in whom these conservative measures fail—specifically, when tenacious distal secretions, mucus plugging, or lobar atelectasis persist despite optimal standard care. Routine daily bronchoscopy is not recommended for unselected mechanically ventilated AECOPD patients.

Conventional suction catheters are effective for clearing proximal airway secretions in the majority of mechanically ventilated patients. This proximal reach fails, however, for distal tenacious secretions or segmental mucus plugs—the precise problems that bronchoscopy is designed to address. FOB provides targeted clearance under direct vision for these cases, serving as a supplemental rescue strategy rather than a routine replacement for conventional suction.

Qiao et al. (10) conducted a randomized controlled trial comparing daily bronchoscopic suction with conventional suction in AECOPD patients receiving sequential invasive-noninvasive ventilation. The bronchoscopy group demonstrated earlier PIC window appearance, shorter invasive ventilation duration, total ventilation duration, and hospital stay, along with lower reintubation rates, VAP incidence, and mortality, and higher weaning success rates (all *P* < 0.05). This benefit was demonstrated in a selected cohort of patients with severe, refractory airway secretion burden; these favorable results should not be interpreted as endorsing routine daily bronchoscopy, which increases procedural risk and resource consumption without proven incremental benefit in lower-severity populations.

Tang et al. (11) performed a meta-analysis of randomized controlled trials confirming that FOB reduces VAP incidence in invasively ventilated patients compared with general sputum suction. Ellekjaer et al. (12) conducted a systematic review of therapeutic bronchoscopy for acute respiratory failure; however, the evidence base requires careful scrutiny (see below). Nannapaneni et al. (13) reported that implementing bronchoscopy in trauma ICU admission protocols markedly decreased VAP rates in trauma patients; the generalizability of these findings to intubated AECOPD patients requires confirmation.

However, these findings should be interpreted with caution. Ellekjaer et al. (12) conducted a systematic review of therapeutic bronchoscopy versus standard care in critically ill patients with acute respiratory failure, including five trials (*n* = 212) that were all judged as having high risk of bias. Their meta-analysis found no statistically significant difference in all-cause mortality (TSA-adjusted RR 0.39; 95% CI 0.14–1.07) or ICU length of stay. Although a shorter duration of mechanical ventilation was suggested by conventional meta-analysis, trial sequential analysis indicated that only 42% of the required information size had been accrued, signaling a high risk of random error. The authors concluded that the evidence base for therapeutic bronchoscopy in acute respiratory failure remains very low, with no firm evidence for benefit or harm. This underscores that the current evidence base for therapeutic bronchoscopy in acute respiratory failure remains very low quality, and routine bronchoscopic interventions should not be recommended without careful patient selection. These studies were conducted in mixed populations of critically ill patients rather than specifically in intubated AECOPD patients, and their generalizability to intubated AECOPD patients therefore requires confirmation.

#### 2.2.2 Bronchoalveolar lavage

For patients with large mucus plugs or highly viscous secretions, BAL using 37 °C normal saline (10–20 mL per aliquot, total ≤100 mL) helps liquefy and mobilize retained secretions. BAL specimens also serve dual diagnostic-therapeutic purposes (1).

#### 2.2.3 Atelectasis management

Retained secretions frequently cause lobar or segmental atelectasis in mechanically ventilated patients. Kreider and colleagues (14) reviewed the role of bronchoscopy for atelectasis in the ICU, noting that it is one of the most common indications despite limited dedicated research. Athish et al. (15) described a case series of postoperative patients with acute lung atelectasis due to mucus plugging successfully managed with bronchoscopic clearance combined with chest physiotherapy.

#### 2.2.4 Facilitation of weaning: overview

The pulmonary infection control (PIC) window (肺部感染控制窗) concept, proposed by Chinese investigators, refers to the period during AECOPD management when infection is controlled—sputum volume decreases, secretion consistency changes from purulent to mucoid, and systemic signs of infection abate. This concept is widely used in Chinese weaning practice but has not been externally validated or incorporated into international guidelines. The strategy of early extubation at the PIC window followed by noninvasive ventilation (NIV)- termed sequential invasive-noninvasive ventilation- has been evaluated in several meta-analyses conducted in Chinese patient populations; these findings have not been replicated outside China (16–18). Although this concept has been widely adopted in Chinese weaning protocols, its objective criteria have not been externally validated, and the absence of standardized thresholds limits inter-institutional reproducibility.

For context, spontaneous breathing trial (SBT) is the current international standard for assessing extubation readiness in mechanically ventilated patients, and its role in AECOPD weaning is well established (19). SBT failure remains common in AECOPD nonetheless, often driven by retained secretions, respiratory muscle weakness, and dynamic hyperinflation. In this context, bronchoscopy-assisted clearance at the PIC window is not proposed as a replacement for SBT, but as a complementary strategy that addresses the secretion-related component of weaning failure—a factor that SBT alone does not directly address. The proposed PIC window is intended to identify the timing of infection resolution, addressing the infectious prerequisite for weaning; SBT evaluates respiratory muscle endurance and ventilatory capacity, assessing the physiological feasibility of extubation. These two assessments address distinct dimensions of weaning readiness. The practical challenge—and one that remains unanswered—is how to sequence these approaches in individual patients. The optimal approach may combine the structured SBT-based evaluation of respiratory drive and muscle strength with PIC window-guided secretion management, though this integrated strategy has not been formally tested.

Specific evidence and procedural considerations for pre-extubation bronchoscopy are detailed in Section 3.3.

## 3 Procedural timing: a preliminary, hypothesis-generating framework

**Figure 2:**
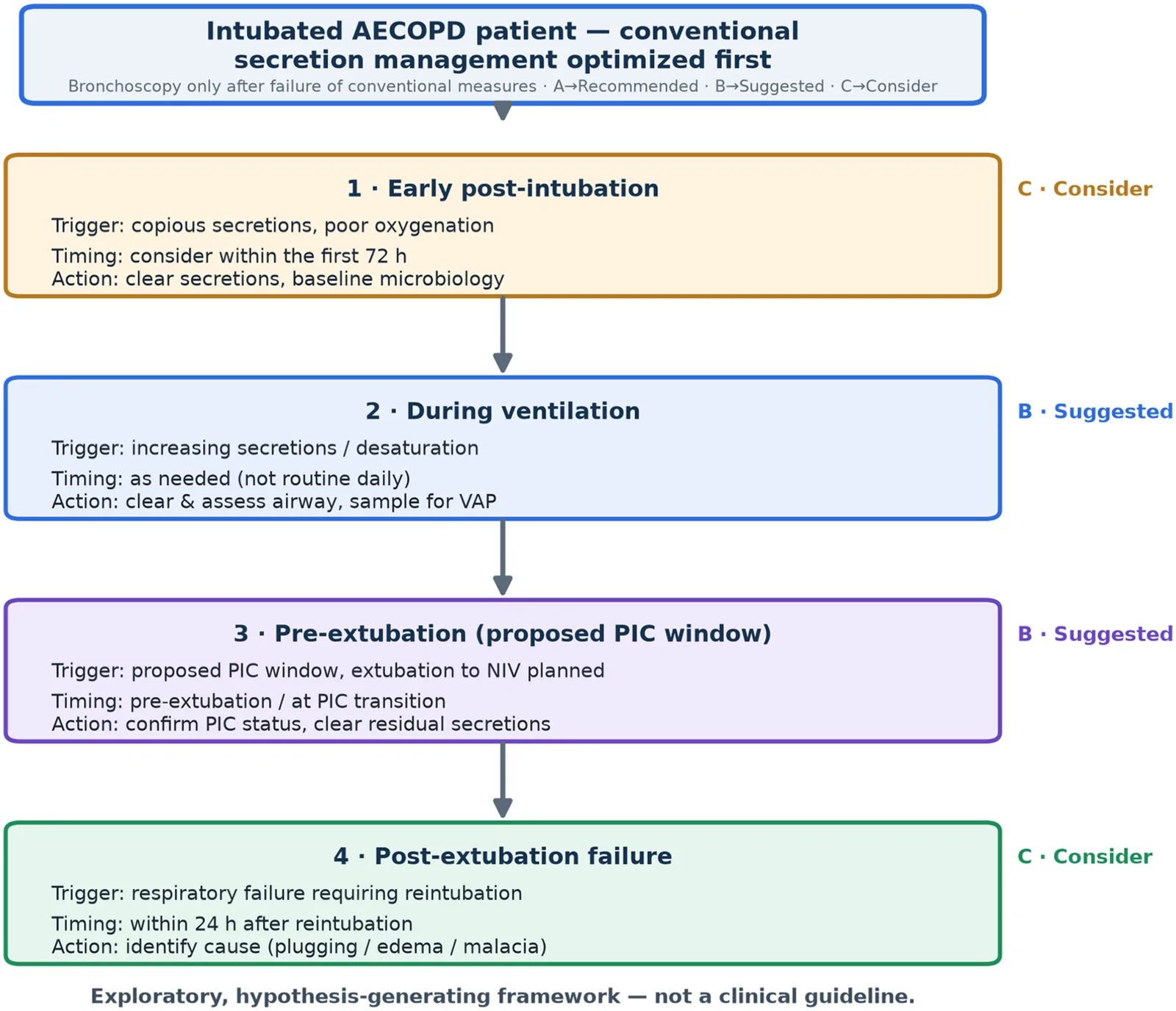
Proposed timing algorithm for bedside bronchoscopy in intubated AECOPD patients. Each branch indicates the clinical trigger, suggested timing, proposed intervention, and the corresponding certainty of evidence (A/B/C) and recommendation strength.

No guideline currently provides quantitative recommendations for when bedside bronchoscopy should be performed in mechanically ventilated AECOPD patients. Based on available indirect evidence and clinical reasoning, we propose a four-scenario framework (Table 1), which is also presented as a clinical algorithm in Figure 2. This framework is exploratory and hypothesis-generating; it is not a clinical guideline, and none of the following scenarios should be interpreted as guideline-endorsed standards. It is intended as a clinical reference for timing decisions and as a testable structure for future investigation.

**Table 1:** Proposed framework for bedside bronchoscopy timing in different clinical scenarios.

Clinical scenario	Suggested timing	Purpose	Procedural caution	Certainty of evidence (A/B/C)	Strength of recommendation
Massive secretions at admission, poor oxygenation	May be considered within the first 72 h	Clear initial secretions, obtain baseline microbiology	Assess hemodynamic stability	C (indirect evidence; clinical experience)	Consider
Increasing secretions or unexplained desaturation during MV	As needed (not routine daily)	Clear secretions, assess airway, sample for suspected VAP	Avoid unnecessary procedures	B (meta-analysis; retrospective before-after study) (11, 20)	Suggested
PIC window achieved, planning extubation to NIV	Pre-extubation/at the proposed PIC transition	May be considered to confirm PIC status and clear residual secretions	Limit procedure duration	B (single-center RCT, conducted in China) (16, 21)	Suggested
Post-extubation respiratory failure requiring reintubation	Within 24 h after reintubation	Identify cause (mucus plugging, edema, tracheomalacia)	Targeted intervention based on etiology	C (expert opinion)	Consider

Certainty of evidence and recommendation strength follow the same predefined three-tier scheme as Table 2. This framework is exploratory and hypothesis-generating, not a clinical guideline; none of these scenarios should be interpreted as guideline-endorsed standards. The PIC window is a concept used in Chinese practice that has not been externally validated; entries referencing the PIC window should be interpreted as hypothesis-generating. See also the clinical algorithm in Figure 2.

**Table 2:** Summary of clinical applications of bedside bronchoscopy in mechanically ventilated AECOPD patients.

Application category	Specific application	Certainty of evidence (A/B/C)	Recommendation strength	References
Diagnostic	Airway assessment (mucosa, secretions, anatomy)	C (expert opinion)	Consider	(1, 2)
Diagnostic	Microbiological sampling (BAL/PSB)	A (systematic review/meta-analysis; guideline-supported)	Recommended	(3, 4, 22)
Diagnostic	Differential diagnosis	C (expert opinion)	Consider	(2)
Therapeutic	Airway secretion clearance	B (single-center RCT; meta-analysis)	Suggested	(10, 11, 13)
Therapeutic	Bronchoalveolar lavage	C (limited evidence)	Consider	(1)
Therapeutic	Atelectasis management	C (case series)	Consider	(14, 15)
Therapeutic	Weaning facilitation (PIC window)	B (single-center RCT, conducted in China)	Suggested	(16, 21)

Certainty of evidence follows a predefined three-tier scheme: A = high-quality, multi-center randomized controlled trials with low risk of bias, or high-quality systematic reviews/meta-analyses; B = single-center, small, or high-risk-of-bias randomized controlled trials, or large prospective observational studies; C = retrospective observational studies, case series, indirect evidence, or expert opinion. Recommendation strength maps as: A → Recommended; B → Suggested; C → Consider. This is an author-generated categorization and does not follow formal GRADE methodology; it is intended as a rapid reference for clinical readers. The PIC window is a concept used in Chinese practice that has not been externally validated; entries referencing the PIC window should be interpreted as hypothesis-generating.

### 3.1 Early post-intubation period

Bronchoscopy may be considered within the first 72 h in patients with copious airway secretions, audible rhonchi, and poor oxygenation at presentation, provided conventional measures have been optimized. The goal is to clear accumulated secretions, collect baseline microbiological specimens, and assess the airway. Scala et al. (23) demonstrated the safety and feasibility of early FOB in decompensated COPD patients with community-acquired pneumonia receiving NIV support; these data are therefore indirect for intubated patients. Whether this safety profile holds for intubated patients—who lack native airway reflexes and often have hyperinflation—remains to be determined. The optimal timing within the first 72 h is an open question. Potential triggers that may prompt consideration of bronchoscopy at this stage include: FiO₂ requirement exceeding 0.6, suctioned secretion volume >30 mL/24 h, or new lobar atelectasis on chest radiography despite optimal conservative airway management.

### 3.2 During mechanical ventilation (as needed)

Indications include: (1) increasing airway secretions poorly responsive to conventional suction; (2) unexplained oxygenation deterioration or ventilatory impairment; (3) suspected VAP requiring microbiological sampling (3, 22); (4) chest imaging suggesting new atelectasis (14). These indications may be incorporated into a procedural checklist to minimize unnecessary bronchoscopies and practice variation.

Duesberg et al. (20) provided a valuable experience: implementing evidence-based indications for on-call bronchoscopy reduced after-hours procedures by 83.6% (from 794 to 130 procedures) without worsening patient outcomes. This demonstrates that appropriate indication refinement optimizes resource allocation while ensuring timely intervention for those who need it.

### 3.3 Pre-extubation (at the proposed PIC window)

No study has specifically evaluated bronchoscopy performed exclusively at the moment of extubation. When patients reach the proposed PIC window and are being considered for extubation to NIV, bronchoscopy performed during this transition offers three potential benefits: (1) direct visual confirmation that the airway is indeed clean (rather than relying solely on clinical signs); (2) clearance of residual secretions to reduce post-extubation mucus plugging and reintubation risk; (3) assessment of airway patency and mucosal integrity.

The following discussion is organized around the PIC window concept as an exploratory framework. This concept, while supported by several Chinese studies, has not been adopted in international guidelines and should be viewed as hypothesis-generating rather than as an established standard.

The proposed PIC window criteria (decreased sputum volume, change from purulent to mucoid consistency, abatement of systemic infection signs) indicate systemic infection control, not necessarily complete airway dryness. Residual secretions in segmental or subsegmental bronchi may persist after systemic infection has resolved and can obstruct the airway after extubation. Bronchoscopy at this transition therefore serves a dual function: it confirms that the airway is safe for extubation and removes residual material that may not be reachable by closed suction.

**Figure 3:**
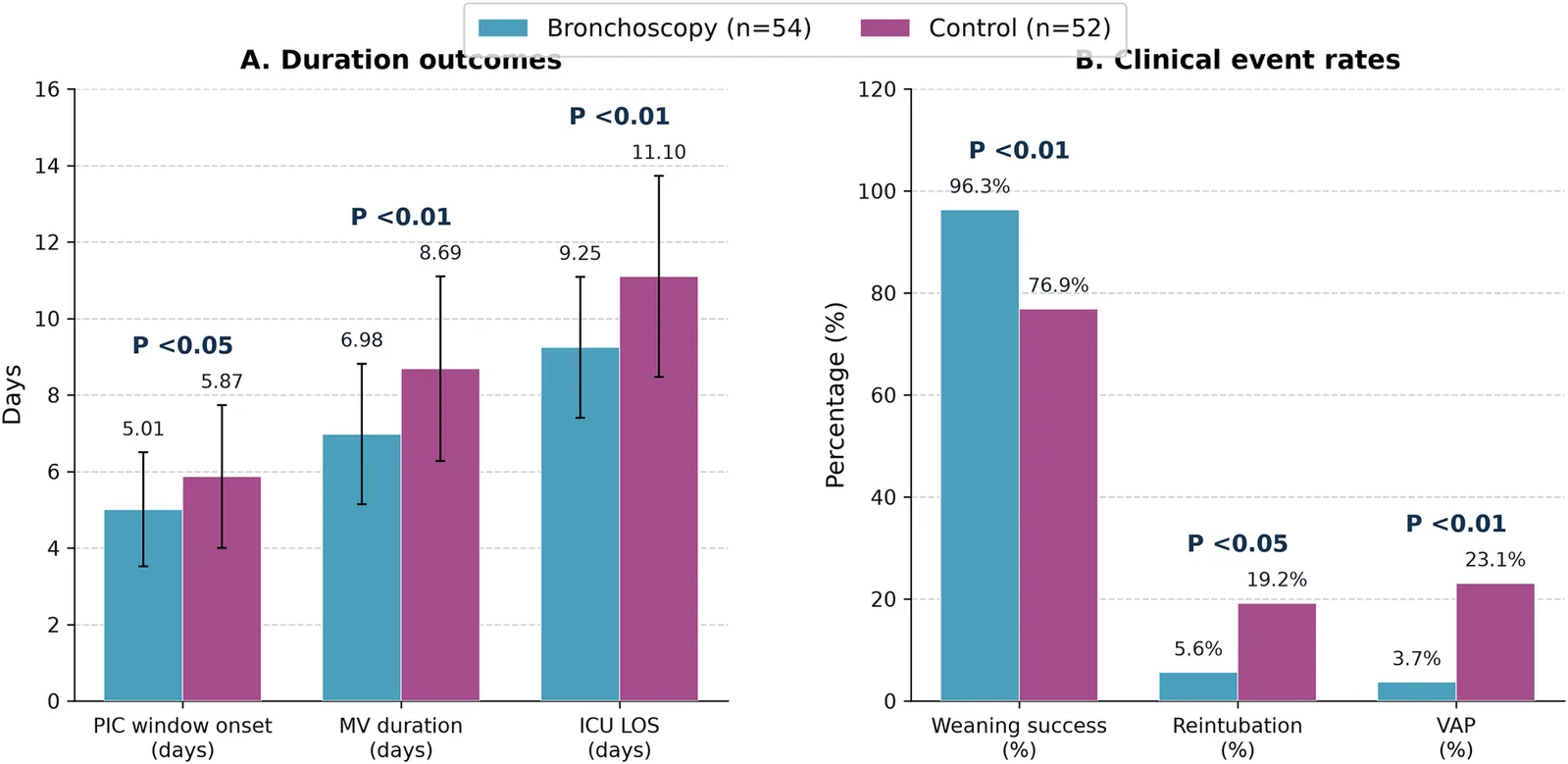
Comparison of clinical outcomes between bronchoscopy and control groups. **(A)** Duration outcomes: PIC window onset, mechanical ventilation duration, and ICU length of stay. **(B)** Clinical event rates: first-attempt weaning success, reintubation rate, and VAP incidence. Data are presented as mean with SD or percentage. *P* values are from independent t-test or chi-square test. Scenario 3 (proposed PIC window) is graded B on the basis of a single-center randomized trial (Song et al., 2012) with significant limitations and should be interpreted as hypothesis-generating. This algorithm is an exploratory, hypothesis-generating framework, not a clinical guideline; bronchoscopy is considered after failure of conventional secretion management. Certainty of evidence: A = high-quality, multi-center randomized controlled trials with low risk of bias, or high-quality systematic reviews/meta-analyses; B = single-center, small, or high-risk-of-bias randomized controlled trials, or large prospective observational studies; C = retrospective observational studies, case series, indirect evidence, or expert opinion. Recommendation strength: A → Recommended; B → Suggested; C → Consider. *P* values are reported as threshold values as in the original publication (Song et al., 2012) (21).

Song et al. (21) conducted a prospective randomized study of FOB-assisted sputum suction and BAL in AECOPD patients during the sequential weaning transition at the PIC window—a time point that in clinical practice typically coincides with the pre-extubation phase. The study enrolled 106 AECOPD patients with respiratory failure (bronchoscopy group *n* = 54, control *n* = 52). Results are presented in Table 3 and Figure 3.

**Table 3.** Clinical outcomes from Song et al. (21).

**Parameter**	**Bronchoscopy group (*n* = 54)**	**Control group (*n* = 52)**	***P* value**
PIC window onset (days)	5.01 ± 1.49	5.87 ± 1.87	<0.05
Mechanical ventilation duration (days)	6.98 ± 1.84	8.69 ± 2.41	<0.01
ICU length of stay (days)	9.25 ± 1.84	11.10 ± 2.63	<0.01
First-attempt weaning success rate (%)	96.30%	76.92%	<0.01
Reintubation rate (%)	5.56%	19.23%	<0.05
VAP incidence	3.70%	23.07%	<0.01

Note: *P* values are reported exactly as in the original publication (Song et al., 2012), which reported threshold values (*P* < 0.05 / *P* < 0.01) rather than exact P values. The PIC window is a concept used in Chinese practice that has not been externally validated; these single-center outcome data should be interpreted as hypothesis-generating.

The magnitude of these differences warrants cautious interpretation. The 23.07% VAP rate in the control group is higher than contemporary benchmarks reported from well-established VAP prevention programs, suggesting that baseline care quality in the control arm may have differed from current standards. The exceptionally low VAP rate (3.70%) in the bronchoscopy group—an absolute risk reduction of 19.37% corresponding to a number needed to treat of approximately 5—exceeds what would be expected from bronchoscopy alone in contemporary ICUs with standard VAP prevention bundles. This suggests that unmeasured confounders, including differences in nursing care intensity, surveillance bias, or baseline infection control practices, may have contributed to the observed effect. These results should be considered hypothesis-generating rather than definitive, and replication in modern, multi-center settings is needed before clinical recommendations based on these specific effect sizes can be made.

Li et al. (24) conducted an indirect comparison meta-analysis suggesting that the PIC window strategy may be superior to SBT-failure-triggered NIV conversion in reducing invasive ventilation duration.

### 3.4 Post-extubation failure/after reintubation

When a patient develops respiratory failure after extubation requiring reintubation, bronchoscopy can help distinguish among mucus re-accumulation, laryngeal edema, and tracheobronchial collapse, each of which requires a different management strategy. This scenario lacks systematic clinical investigation and recommendations are based on clinical experience.

## 4 Standardized management

### 4.1 Pre-procedural preparation

#### 4.1.1 Risk assessment

Li et al. (25) conducted a systematic review of 18 studies enrolling 2000 COPD patients undergoing diagnostic bronchoscopy. The overall major complication rate was 4.3% (95%CI 2.2%–8.2%), with higher rates during exacerbations (7.8%) versus stable disease (4.5%). The BTS guideline (4) reported complication rates of 5% in patients with severe COPD (defined in the study cited by the guideline as FEV1/FVC <50%, or FEV1 <1 L with FEV1/FVC <69%) compared with 0.6% in patients with normal lung function; the guideline separately recommends pre-bronchoscopy arterial blood gas analysis in patients with FEV1 <40% predicted and/or SaO2 <93%. Sedation is associated with increased risk, as demonstrated by a meta-analysis of 18 trials (25). Whether obesity or a high STOP-BANG score—validated predictors in elective bronchoscopy (26, 27)—carries the same weight in mechanically ventilated ICU patients is less certain. However, the applicability of these predictors to mechanically ventilated AECOPD patients—who often have hypercapnia, hemodynamic instability, and dynamic hyperinflation—has not been specifically validated.

#### 4.1.2 Ventilator adjustment

FiO2 should be increased to 100% prior to the procedure. PEEP may be increased to reduce alveolar collapse during bronchoscope insertion.

#### 4.1.3 Sedation and oxygenation strategies

Pelaia et al. (28) systematically reviewed oxygenation strategies during FOB—data derived predominantly from non-intubated or non-ICU populations—finding high-flow nasal cannula (HFNC) superior to conventional oxygen therapy in patients with moderate-to-severe acute respiratory failure; the generalizability of these findings to intubated AECOPD patients requires confirmation. For COPD patients with hypercapnia, NIV-assisted bronchoscopy may be the optimal approach (2, 29–31). Longhini et al. (32) further reviewed the physiologic rationale for HFNC during FOB in critically ill patients.

Several novel sedative agents have been investigated for bronchoscopy. Pastis et al. (33) reported that remimazolam achieved 80.6% procedural success versus 4.8% for placebo in a multicenter RCT; however, this study was conducted in elective outpatient bronchoscopy, and its results may not directly generalize to mechanically ventilated ICU patients. Matthes et al. (34) conducted a systematic review of sedation safety during flexible bronchoscopy, emphasizing the importance of provider experience and monitoring. However, these data were derived predominantly from outpatient or non-ICU populations undergoing elective diagnostic bronchoscopy. Caution is warranted when extrapolating these findings to mechanically ventilated AECOPD patients with hypercapnia, hemodynamic instability, or severe airflow obstruction, as this subgroup was underrepresented in the original trials.

#### 4.1.4 Equipment selection

The Chinese Medical Association respiratory branch published expert consensus on single-use (disposable) flexible bronchoscopes, endorsing their utility in high-infection-risk settings, emergency procedures, and resource-limited hospitals (35). Marshall et al. (36) reported ICU experience with disposable bronchoscopes in Singapore, with common indications including percutaneous tracheostomy guidance (44.6%), microbiological sampling (24.1%), airway inspection, and toilet.

### 4.2 Intraprocedural technique

Insert the bronchoscope through the swivel adapter on the endotracheal tube or dedicated port, allowing uninterrupted mechanical ventilation. Continuous monitoring of SpO2, heart rate, blood pressure, and end-tidal CO2 (EtCO2) is essential. EtCO2 monitoring is particularly important in COPD patients, as standard pulse oximetry cannot detect developing hypercapnia. Keep suction pressure ≤13.3 kPa (100 mmHg), and procedure duration should generally be limited to 15–20 min.

### 4.3 Complications

Transient hypoxemia is the most common complication. Hypercapnia requires special attention in COPD patients. Scala (2) emphasized that expected procedural benefits must be balanced against cardiopulmonary complications, bleeding, and pneumothorax risk. Li et al. (25) confirmed that overall complication rates for diagnostic bronchoscopy in COPD are acceptable. Patolia et al. (37) reviewed bronchoscopy safety in both intubated and non-intubated ICU patients, noting that careful patient selection and experienced operators make the difference between a safe procedure and a complicated one. In patients with severe AECOPD requiring high FiO2 (>0.6), hemodynamic instability, or profound hypercapnia, the risk of procedural hypoxemia, hypercapnic respiratory acidosis, and hemodynamic decompensation is substantially elevated. In such cases, the anticipated benefits of bronchoscopy should be weighed particularly carefully against these heightened risks, and alternative approaches should be considered when feasible.

### 4.4 Post-procedural management

FiO2 should be gradually reduced to pre-procedural levels while monitoring oxygenation and hemodynamics. Send BAL specimens for microbiological and cytological analysis.

### 4.5 Special considerations for secondary hospital ICUs (Box 1)

Box 1 **Expert Practice Considerations for Resource-Limited Settings**

**Table:** 

*The following suggestions are derived from the authors’ clinical experience in Chinese secondary hospital ICUs and should be interpreted as expert opinion rather than evidence-based recommendations. What follows is a distillation of practical lessons learned in resource-constrained settings—where access to current-generation ventilators may be limited, disposable scopes represent a significant budget consideration, and the on-call operator may have limited procedural experience (in some settings fewer than 50 procedures). All thresholds and procedural parameters mentioned in this box are derived from the authors’ institutional experience and are not evidence-based. Readers are encouraged to evaluate these suggestions in the context of their own institutional resources and patient populations.* **Equipment.** In ICUs with lower procedure volumes, the per-procedure amortized cost of a reusable bronchoscope (including purchase, reprocessing, repair, and storage) may approach or exceed the unit cost of disposable bronchoscopes. The Chinese expert consensus (35) has highlighted the potential advantages of disposable scopes in resource-limited settings, but formal cost-effectiveness analyses in secondary hospital ICUs remain unavailable. **Personnel training.** Tertiary centers typically require operators to have completed 3 + years of bronchoscopy experience, and competency frameworks for ICU bronchoscopy have been proposed (38). In settings where training duration may be shorter, independent operation may be authorized based on documented competency in airway handling, suction technique, complication management, and recognition of anatomical variants under direct supervision, rather than on a fixed procedural count. Relative contraindications to sole-operator status may be defined, such as FiO2 requirements >80% to maintain SpO2 > 90% or hemodynamic instability requiring continuous vasoactive support. **Technical prerequisites.** Section 4.1 describes NIV-assisted bronchoscopy as a potential approach for hypercapnic COPD patients. In secondary hospitals, this approach requires verification that the available ventilator supports NIV mode with adequate leak compensation and that staff are trained in synchronized NIV delivery during bronchoscopy. Where these conditions are not met, bronchoscopy may instead be performed under invasive mechanical ventilation settings (increased FiO2, PEEP) rather than NIV. **Monitoring alternatives.** Some secondary ICUs may lack EtCO2 monitoring. In such settings, we suggest: (1) limiting procedure duration to 10 minutes or less; (2) extending post-procedural SpO2 monitoring to 30 minutes to detect delayed desaturation; (3) obtaining arterial blood gas analysis 1 hour post-procedure in patients with known baseline hypercapnia (PaCO2 > 50 mmHg).

## 5 Evidence gaps and limitations

1. No multicenter trial has directly compared timing strategies. The four-scenario framework rests on indirect evidence and clinical reasoning—a pragmatic RCT with ventilator-free days as the primary endpoint would be a logical next step. A pragmatic design randomizing AECOPD patients to early (within 24 h of intubation) versus PIC-window bronchoscopy, with ventilator-free days at day 28 as the primary endpoint, would address this gap directly. Such a trial should recruit from both tertiary and secondary hospital ICUs to improve generalizability.
2. The PIC window currently lacks standardized operational criteria. Reduced sputum volume, improved secretion character, and falling infection markers are clinically sensible but not standardized. Based on our synthesis of the most consistently reported PIC-window descriptors in the Chinese literature—selected because they are measurable at the bedside with widely available tools—and the authors’ clinical experience, we propose the following provisional, hypothesis-generating criteria for consideration in future research: sputum volume <30 mL/24 h by serial suction logging, conversion from purulent to mucoid secretion on visual inspection, and a decline in procalcitonin to <0.25 µg/L or by >80% from peak. These thresholds are suggested solely as a starting point for standardization and require prospective validation against clinical outcomes; they should not be interpreted as validated clinical thresholds or as recommendations for current practice. Whether dynamic compliance trends, procalcitonin kinetics, or mini-BAL cell profiles could sharpen the definition is a testable question (16). A prospective multicenter study correlating procalcitonin kinetics and mini-BAL neutrophil percentage with clinically defined PIC window status could provide the first objective operational criteria for this concept.
3. Sample sizes remain modest. Song et al. (21) enrolled 106 patients in a single-center randomized trial conducted in 2012; although this remains the largest study to date, its findings require replication in contemporary multicenter cohorts.
4. Data from secondary hospitals are scarce in the published literature; nearly all available studies derive from tertiary centers. Prospective observational studies documenting complication rates and operator learning curves in secondary ICUs are needed to assess external validity.

### 5.1 Strengths and limitations

This review offers several strengths: a clinically grounded framework for procedural timing, dedicated attention to secondary hospital settings that are underrepresented in the literature, and all cited references were verified against PubMed/Crossref records. To our knowledge, it is the first review to synthesize the available evidence on bedside bronchoscopy timing across the full trajectory of mechanical ventilation in AECOPD.

Several limitations must be acknowledged. First, this is a narrative review by design; the absence of a formal systematic search protocol, predefined inclusion/exclusion criteria, or PRISMA flowchart is inherent to this review type rather than a methodological limitation. Nevertheless, we acknowledge that this approach introduces potential selection bias in the cited literature, and we cannot exclude the possibility that relevant studies were missed. Second, the core evidence for bronchoscopy-assisted weaning (21) derives from a single-center RCT from 2012 with a relatively small sample size and effect sizes that may be influenced by the prevailing care standards of that era. Third, the PIC window concept, while supported by several Chinese meta-analyses, has not been adopted in international respiratory or critical care guidelines; the timing framework proposed here is therefore a hypothesis-generating structure rather than a validated protocol. Fourth, nearly all evidence originates from tertiary centers in China, and generalizability to other healthcare systems and resource settings is uncertain. Fifth and finally, the section on secondary hospital ICUs (Section 4.5) is based on clinical experience rather than published data and should be interpreted accordingly. Sixth, the BTS bronchoscopy guideline cited as reference (4) was published in 2013; while it remains the most comprehensive guideline available on this topic, readers should be aware of its age and consult newer evidence where available.

## 6 Conclusions

Bedside bronchoscopy is a useful adjunct in mechanically ventilated AECOPD patients for airway assessment, pathogen-directed sampling, secretion clearance, and weaning facilitation. Song et al.'s (21) single-center RCT reported potential benefits for bronchoscopy-assisted weaning at the PIC window, but these results require replication in contemporary multi-center studies before they can be considered definitive. The PIC window concept offers a clinically intuitive but unvalidated framework for weaning timing in AECOPD; it has not been adopted in international guidelines and should be viewed as complementary to, not a replacement for, SBT-based weaning protocols.

Based on the available evidence, we propose a preliminary four-scenario timing framework covering early post-intubation, during mechanical ventilation, pre-extubation, and post-extubation failure scenarios, with special attention to operational realities in secondary hospital ICUs. This framework is hypothesis-generating and requires prospective validation before it can inform clinical practice recommendations. Future research should prioritize a pragmatic multicenter randomized trial comparing early versus PIC-window bronchoscopy, with ventilator-free days as the primary endpoint. Such a trial should be adequately powered and include both tertiary and secondary hospital ICUs to ensure generalizability.

## Data Availability

The original contributions presented in the study are included in the article/Supplementary Material, further inquiries can be directed to the corresponding author.
